# Supplementary material for: Multi-layered ecological interactions determine growth of clinical antibiotic-resistant strains within human microbiomes
Source: Nat Commun. 2025 Nov 4;16:9733. doi: 10.1038/s41467-025-64714-2 (PMC12586700; doi:10.1038/s41467-025-64714-2)
Supplement: Supplementary file 2 — Reporting summary [file 41467_2025_64714_MOESM2_ESM.pdf]

nature portfolio

Corresponding author(s): Ricardo Leon-Sampedro

Last updated by author(s): Jul 30, 2025

Reporting Summary

Nature Portfolio wishes to improve the reproducibility of the work that we publish. This form provides structure for consistency and transparency in reporting. For further information on Nature Portfolio policies, see our Editorial Policies and the Editorial Policy Checklist.

Statistics

For all statistical analyses, confirm that the following items are present in the figure legend, table legend, main text, or Methods section.

n/a	Confirmed	
☐	☒	The exact sample size (n) for each experimental group/condition, given as a discrete number and unit of measurement
☐	☒	A statement on whether measurements were taken from distinct samples or whether the same sample was measured repeatedly
☐	☒	The statistical test(s) used AND whether they are one- or two-sided<br>*Only common tests should be described solely by name; describe more complex techniques in the Methods section.*
☐	☒	A description of all covariates tested
☐	☒	A description of any assumptions or corrections, such as tests of normality and adjustment for multiple comparisons
☐	☒	A full description of the statistical parameters including central tendency (e.g. means) or other basic estimates (e.g. regression coefficient) AND variation (e.g. standard deviation) or associated estimates of uncertainty (e.g. confidence intervals)
☐	☒	For null hypothesis testing, the test statistic (e.g. F, t, r) with confidence intervals, effect sizes, degrees of freedom and P value noted<br>*Give P values as exact values whenever suitable.*
☒	☐	For Bayesian analysis, information on the choice of priors and Markov chain Monte Carlo settings
☒	☐	For hierarchical and complex designs, identification of the appropriate level for tests and full reporting of outcomes
☐	☒	Estimates of effect sizes (e.g. Cohen's d, Pearson's r), indicating how they were calculated

Our web collection on statistics for biologists contains articles on many of the points above.

Software and code

Policy information about availability of computer code

Data collection	Distances between genomes were established using Mash v2.0 and a phylogeny was constructed with mashtree v0.33. The tree was represented with midpoint root using the phytools package in R and visualised using the iTOL tool. To determine the phylotype of each genome, we used the ClermonTyping tool.
Data analysis	- From the growth data obtained from the plate reader (Tecan NanoQuant Infinite M200 Pro), measuring optical density (OD600), we extracted the maximum growth rate (μ), maximum optical density (ODmax), and area-under-the-curve (AUC) for each well using growthrates and flux packages in R.<br>- To determine the distribution of the E. coli isolates across the phylogeny of the species, we obtained 1334 assemblies of E. coli complete genomes from the RefSeq database (https://www.ncbi.nlm.nih.gov/assembly). Distances between genomes were established using Mash v2.0 and a phylogeny was constructed with mashtree v0.33. The tree was represented with midpoint root using the phytools package in R and visualised using the iTOL tool. To determine the phylotype of each genome, we used the ClermonTyping tool.

For manuscripts utilizing custom algorithms or software that are central to the research but not yet described in published literature, software must be made available to editors and reviewers. We strongly encourage code deposition in a community repository (e.g. GitHub). See the Nature Portfolio guidelines for submitting code & software for further information.

Data

Policy information about availability of data

All manuscripts must include a data availability statement. This statement should provide the following information, where applicable:

- Accession codes, unique identifiers, or web links for publicly available datasets
- A description of any restrictions on data availability
- For clinical datasets or third party data, please ensure that the statement adheres to our policy

- Sequences of the 16S rRNA amplicons from the 90 microbiome samples and the genomes of the three resident E. coli strains are uploaded to the NCBI and ENA (European Nucleotide Archive) and will be accessible through projects PRJNA1179737 and PRJEB80311..
- All other data supporting the results of this study will be made available through Figshare under the following DOI: 10.6084/m9.figshare.29669927

Research involving human participants, their data, or biological material

Policy information about studies with human participants or human data. See also policy information about sex, gender (identity/presentation), and sexual orientation and race, ethnicity and racism.

Reporting on sex and gender	This information was not collected.
Reporting on race, ethnicity, or other socially relevant groupings	This information was not collected.
Population characteristics	This information was not collected.
Recruitment	Stool samples were collected from healthy subjects at the Department of Environmental Systems Science, ETH Zürich, Switzerland, on 17 March 2022 and 24 May 2022 (approved by the Ethics Commission of ETH Zürich, number EK-2020-N-150). Inclusion criteria were: over 18 years old, healthy, not obese, not recovering from surgery, not taken antibiotics in the past six months, and not tested positive for SARS-CoV-2 in the past two months.
Ethics oversight	Ethics Commission of ETH Zürich, number EK-2020-N-150

Note that full information on the approval of the study protocol must also be provided in the manuscript.

Field-specific reporting

Please select the one below that is the best fit for your research. If you are not sure, read the appropriate sections before making your selection.

☐ Life sciences ☐ Behavioural & social sciences ☒ Ecological, evolutionary & environmental sciences

For a reference copy of the document with all sections, see nature.com/documents/nr-reporting-summary-flat.pdf

Ecological, evolutionary & environmental sciences study design

All studies must disclose on these points even when the disclosure is negative.

Study description	We used replicated anaerobic microcosms to track the population growth of four clinical strains of antibiotic-resistant E. coli in gut microbiome samples from healthy humans (Fig. 1A&B; Table S1), aiming to identify the factors and interactions that drive their invasion. We hypothesized that different strains within the same species would exhibit different interactions with resident microbiota, leading to variable invasion outcomes. To test this, we used multiple clinical strains and a controlled design with uninvaded microcosms to assess how growth performance and interactions with resident microbiota varied among incoming strains, potentially revealing mechanisms linked with colonization success. Each strain belongs to one of the four most abundant phylogroups in humans and to different sequence types (STs) and carried a different clinically relevant resistance plasmid (encoding Extended-Spectrum β-Lactamases -ESBLs- and carbapenemases) (Table S2). To prepare anaerobic cultures of each focal strain prior to inoculation into microcosms, we picked 72 randomly-selected colonies (18 per focal strain) into 72 Hungate tubes (VWR, Schlieren, Switzerland) containing anaerobic LB (Sigma-Aldrich), and incubated at 37°C overnight. We then used these 72 independent cultures to inoculate 8 µl of one focal E. coli strain (approximately 1:1,000 v:v dilution) per microcosm. We included three replicate microcosms in each combination of human Donor (1, 2 or 3), focal strain (present or absent), and antibiotic (treated or untreated). In total 180 microcosms ([4 focal strains + Uninoculated] × 3 donors × 3 replicates × 2 antibiotic conditions × 2 timepoints).
Research sample	- Stool samples were collected from healthy subjects at the Department of Environmental Systems Science, ETH Zürich, Switzerland, on 17 March 2022 and 24 May 2022 (approved by the Ethics Commission of ETH Zürich, number EK-2020-N-150). Inclusion criteria were: over 18 years old, healthy, not obese, not recovering from surgery, not taken antibiotics in the past six months, and not tested positive for SARS-CoV-2 in the past two months. Each sample was collected in a 500-ml plastic specimen container (Sigma-Aldrich) and kept anaerobic using one AnaeroGen anaerobic sachet (Thermo Scientific, Basel, Switzerland).<br>- We used four clinical E. coli strains as focal strains (Table S1). These were obtained from hospitalized patients in two different studies at the University Hospital Basel, Switzerland46,47. The four strains belong to four different phylogroups and sequence types

	(STs), and carry different conjugative antibiotic-resistance plasmids (Table S1 and S2). Ec040 and Ec069 carry pESBL15 (IncI, 88.9 kb) and pESBL25 (IncFIA, IncFIB, 131kb) respectively, encoding extended spectrum beta-lactamases (ESBL) of the CTX-M type. Ec131 and Ec744 carry pKPC and pOXA-48 respectively, with resistance genes encoding carbapenemases, a subgroup of beta-lactamases.
Sampling strategy	Stool samples were collected from healthy subjects at the Department of Environmental Systems Science, ETH Zürich, Switzerland, on 17 March 2022 and 24 May 2022, under approval from the Ethics Commission of ETH Zürich (approval number EK-2020-N-150). The sampling process was fully anonymized, ensuring that it is not possible to reconnect a sample back to an individual donor. Inclusion criteria required donors to be over 18 years old, healthy, not obese, not recovering from surgery, not have taken antibiotics in the past six months, and not have tested positive for SARS-CoV-2 in the past two months.<br><br>From a larger pool of donated samples, three samples were randomly selected for the experiment. The decision to use only three samples was based on two factors: (1) the primary goal was to use healthy human gut microbiomes as models/ examples and focus on the effects of the focal strains on these communities rather than compare variability between donors, and (2) the large scale of the experimental design, which included 180 microcosms, necessitated limiting the number of donor samples. Each microcosm represented a combination of human donor (Donor 1, 2, or 3), focal strain (present or absent), and antibiotic treatment (treated or untreated). The final experimental design included three replicate microcosms for each condition, totaling 180 microcosms: ([4 focal strains + uninoculated control] × 3 donors × 3 replicates × 2 antibiotic conditions × 2 timepoints).
Data collection	The data collection procedures are described in detail in the methods section of the manuscript. In summary, stool samples were collected from three healthy human donors and processed within one hour to preserve microbial community integrity. The sampling process was fully anonymized, ensuring that it is not possible to reconnect a sample back to an individual donor. Samples were homogenized, aliquoted, and stored appropriately for downstream analyses. Microcosms were then prepared with combinations of donor microbiomes, four clinical E. coli strains (or uninoculated controls), and antibiotic treatments (treated or untreated).<br><br>Data were collected at two timepoints: shortly after inoculation and after a defined incubation period to capture both immediate and longer-term ecological responses. Microbial community composition was assessed through 16S rRNA amplicon sequencing, while E. coli abundance was quantified using culture-based methods. Sequencing was performed on Illumina MiSeq platform, and data were analyzed through standardized and automated computational pipelines to ensure reproducibility. All experiments included at least three biological replicates per condition, with stringent quality control measures applied throughout the data acquisition process.
Timing and spatial scale	Stool samples were collected from healthy subjects at the Department of Environmental Systems Science, ETH Zürich, Switzerland, on 17 March 2022 and 24 May 2022, under approval from the Ethics Commission of ETH Zürich (approval number EK-2020-N-150).
Data exclusions	No data were excluded from the analysis.
Reproducibility	To ensure reproducibility, we incorporated replicated experimental designs with three biological replicates per condition, including combinations of donor microbiomes, focal strains, and antibiotic treatments. Detailed protocols for all procedures, including stool sample processing, culture conditions, and sequencing workflows, were standardized and meticulously recorded to enable replication. Additionally, experimental conditions such as incubation times, temperatures, and media composition were strictly controlled across all replicates to minimize variability.
Randomization	Randomization of samples was not necessary in this study because the experimental design did not involve comparing treatment effects across a diverse population of donors or conditions. Instead, stool samples from three healthy human donors were used as representative models of the gut microbiome, with a focus on assessing the effects of introducing specific antibiotic-resistant E. coli strains. The primary aim was to study the ecological dynamics within these microbiomes. Additionally, experimental conditions were fully controlled, with all combinations of donor microbiomes, focal strains, and antibiotic treatments systematically replicated in an unbiased manner.
Blinding	Blinding was not necessary for this study because the data acquisition and analysis were based on objective, quantitative measurements (e.g., sequencing data, focal strain abundance) rather than subjective assessments. Additionally, the experimental conditions (donor microbiomes, focal strains, and antibiotic treatments) were predefined and systematically replicated, ensuring unbiased data collection. All analyses were performed using automated pipelines and computational tools, minimizing the potential for bias during data processing and interpretation.

Did the study involve field work? ☐ Yes ☒ No

Reporting for specific materials, systems and methods

We require information from authors about some types of materials, experimental systems and methods used in many studies. Here, indicate whether each material, system or method listed is relevant to your study. If you are not sure if a list item applies to your research, read the appropriate section before selecting a response.

Materials & experimental systems

n/a	Involved in the study
☒	☐ Antibodies
☒	☐ Eukaryotic cell lines
☒	☐ Palaeontology and archaeology
☒	☐ Animals and other organisms
☒	☐ Clinical data
☒	☐ Dual use research of concern
☒	☐ Plants

Methods

n/a	Involved in the study
☒	☐ ChIP-seq
☐	☒ Flow cytometry
☒	☐ MRI-based neuroimaging

Plants

Seed stocks

Report on the source of all seed stocks or other plant material used. If applicable, state the seed stock centre and catalogue number. If plant specimens were collected from the field, describe the collection location, date and sampling procedures.

Novel plant genotypes

Describe the methods by which all novel plant genotypes were produced. This includes those generated by transgenic approaches, gene editing, chemical/radiation-based mutagenesis and hybridization. For transgenic lines, describe the transformation method, the number of independent lines analyzed and the generation upon which experiments were performed. For gene-edited lines, describe the editor used, the endogenous sequence targeted for editing, the targeting guide RNA sequence (if applicable) and how the editor was applied.

Authentication

Describe any authentication procedures for each seed stock used or novel genotype generated. Describe any experiments used to assess the effect of a mutation and, where applicable, how potential secondary effects (e.g. second site T-DNA insertions, mosiacism, off-target gene editing) were examined.

Flow Cytometry

Plots

Confirm that:

☒ The axis labels state the marker and fluorochrome used (e.g. CD4-FITC).

☐ The axis scales are clearly visible. Include numbers along axes only for bottom left plot of group (a 'group' is an analysis of identical markers).

☐ All plots are contour plots with outliers or pseudocolor plots.

☒ A numerical value for number of cells or percentage (with statistics) is provided.

Methodology

Sample preparation

To estimate total microbial abundance in each microcosm, we used flow cytometry. Briefly, we diluted samples by 1:10,000 with sterile and filtered phosphate-buffered saline (PBS) and stained them with SYBR Green (Invitrogen, Thermo Fisher Scientific).

Instrument

We used a Novocyte 2000R (ACEA Biosciences, San Diego, CA, USA), equipped with a 488-nm laser.

Software

NovoExpress software

Cell population abundance

Flow cytometry was used to measure the total bacterial abundance in each microcosm. Samples were stained with SYBR Green I, a nucleic acid dye, to label bacterial cells. Measurements were performed on Novocyte 2000R with gating based on forward and side scatter to distinguish bacterial cells from debris.

The purity of the bacterial population was not applicable, as this study did not involve cell sorting. Instead, gating strategies were designed to exclude non-bacterial particles and background noise, ensuring accurate quantification of bacterial cells. Data acquisition and analysis were conducted using NovoExpress software, and results were expressed as total bacterial cell counts per microcosm.

Gating strategy

The gating strategy was designed to identify bacterial cells while excluding debris and non-bacterial particles. Forward scatter (FSC) and side scatter (SSC) parameters were used to select particles within the size range of bacterial cells, excluding larger debris and smaller background noise. To confirm the presence of nucleic acids, SYBR Green fluorescence intensity was used, with thresholds set to distinguish bacterial cells from non-nucleated particles. Unstained controls were included to determine background fluorescence, and gates were adjusted to avoid false positives.

☒ Tick this box to confirm that a figure exemplifying the gating strategy is provided in the Supplementary Information.
